# Evaluation of three parasite lactate dehydrogenase-based rapid diagnostic tests for the diagnosis of falciparum and vivax malaria

**DOI:** 10.1186/1475-2875-8-241

**Published:** 2009-10-27

**Authors:** Elizabeth A Ashley, Malek Touabi, Margareta Ahrer, Robert Hutagalung, Khayae Htun, Jennifer Luchavez, Christine Dureza, Stephane Proux, Mara Leimanis, Myo Min Lwin, Alena Koscalova, Eric Comte, Prudence Hamade, Anne-Laure Page, François Nosten, Philippe J Guerin

**Affiliations:** 1 Epicentre, Paris, France; 2 Department of Microbiology, Hammersmith Hospital, Imperial College NHS Trust, London, UK; 3 Médecins sans Frontières-Switzerland, Myanmar; 4 Research Institute for Tropical Medicine, Alabang, Muntinlupa City, Philippines; 5 Shoklo Malaria Research Unit, Mae Sot, Thailand; 6 Mahidol-Oxford Tropical Medicine Research Unit (MORU), Mahidol University, Bangkok, Thailand; 7 Centre for Tropical Medicine, Nuffield Department of Clinical Medicine, University of Oxford, UK; 8 Médecins sans Frontières-Switzerland, Geneva, Switzerland; 9 Médecins sans Frontières Malaria Working Group, MSF-UK, UK; 10 Malaria Consortium, London, UK

## Abstract

**Background:**

In areas where non-falciparum malaria is common rapid diagnostic tests (RDTs) capable of distinguishing malaria species reliably are needed. Such tests are often based on the detection of parasite lactate dehydrogenase (pLDH).

**Methods:**

In Dawei, southern Myanmar, three pLDH based RDTs (CareStart™ Malaria pLDH (Pan), CareStart™ Malaria pLDH (Pan, Pf) and OptiMAL-IT^®^)were evaluated in patients presenting with clinically suspected malaria. Each RDT was read independently by two readers. A subset of patients with microscopically confirmed malaria had their RDTs repeated on days 2, 7 and then weekly until negative. At the end of the study, samples of study batches were sent for heat stability testing.

**Results:**

Between August and November 2007, 1004 patients aged between 1 and 93 years were enrolled in the study. Slide microscopy (the reference standard) diagnosed 213 *Plasmodium vivax *(Pv) monoinfections, 98 *Plasmodium falciparum *(Pf) mono-infections and no malaria in 650 cases.

The sensitivities (sens) and specificities (spec), of the RDTs for the detection of malaria were- *CareStart Malaria*™ pLDH (Pan) test: sens 89.1% [CI^95 ^84.2-92.6], spec 97.6% [CI^95 ^96.5-98.4]

*OptiMal-IT^®^*: Pf+/- other species detection: sens 95.2% [CI^95 ^87.5-98.2], spec 94.7% [CI^95 ^93.3-95.8]; non-Pf detection alone: sens 89.6% [CI^95 ^83.6-93.6], spec 96.5% [CI^95 ^94.8-97.7]

*CareStart Malaria*™ pLDH (Pan, Pf): Pf+/- other species: sens 93.5% [CI^95^85.4-97.3], spec 97.4% [95.9-98.3]; non-Pf: sens 78.5% [CI^95^71.1-84.4], spec 97.8% [CI^95 ^96.3-98.7]

Inter-observer agreement was excellent for all tests (kappa > 0.9). The median time for the RDTs to become negative was two days for the CareStart™ Malaria tests and seven days for OptiMAL-IT^®^. Tests were heat stable up to 90 days except for OptiMAL-IT^® ^(Pf specific pLDH stable to day 20 at 35°C).

**Conclusion:**

None of the pLDH-based RDTs evaluated was able to detect non-falciparum malaria with high sensitivity, particularly at low parasitaemias. OptiMAL-IT^® ^performed best overall and would perform best in an area of high malaria prevalence among screened fever cases. However, heat stability was unacceptable and the number of steps to perform this test is a significant drawback in the field. A reliable, heat-stable, highly sensitive RDT, capable of diagnosing all Plasmodium species has yet to be identified.

## Background

Malaria is one of the few diseases for which it is quick and simple to make an accurate biological diagnosis, even in a low-technology setting. Despite this clinical diagnosis is practised widely, even though it has been shown repeatedly to be unreliable [1,2]. In a cross-over study in Zanzibar of 1,887 patients, use of RDTs altered prescribing patterns of antimalarials and anti-bacterials and resulted in improved patient management without increasing costs [3]. Availability of biological diagnosis does not necessarily prevent over-treatment from occurring. In some areas, patients are still likely to receive an antimalarial treatment in the presence of a negative slide or RDT result [4,5].

The choice of diagnostic method in most of the malaria-affected world will be between microscopy and a rapid diagnostic test. Maintaining a high standard of microscopy is challenging and depends on having well-trained experienced technicians, who are not overburdened with slides to read, a continuous supply of good quality staining reagents and appropriately maintained microscopes.

RDTs for malaria are based on the detection of either histidine-rich protein 2 (HRP-2), produced only by *Plasmodium falciparum*, parasite specific lactate dehydrogenase (pLDH) produced by all four species or plasmodium aldolase from the parasite glycolytic pathway, also found in all species. HRP-2 based tests may be misleading in areas of high transmission because they remain positive for a number of days or weeks after an infection, even if treated, thus a positive result with a history of a recently treated infection is difficult to interpret. Another limitation of HRP-2 based tests is their geographically variable sensitivity, attributed to polymorphisms in HRP-2 [6]. Tests based on detection of pLDH or aldolase allow parasite speciation, do not appear to show geographical variability in their ability to detect malaria and revert to negative more quickly than HRP2 based tests, although production of pLDH from gametocytes after elimination of asexual stages means some will stay positive for a number of days [7]. However, to date the sensitivity of these tests under field conditions has been reported frequently as falling below 90% [8,9]. There are concerns about the stability of all types of tests if transportation and storage conditions are not controlled, but pLDH tests appear to be particularly vulnerable [10].

In areas where mixed species infections are common e.g. Asia, Latin America, a reliable test to distinguish between species is needed, since the treatments recommended for falciparum and non-falciparum infections are different. In most parts of the world non-falciparum infections remain susceptible to chloroquine, although chloroquine-resistant vivax malaria has emerged in parts of Indonesia and South America [11,12].

To be adopted in the field a test needs to be >95% sensitive and specific for the detection of falciparum malaria at a parasitaemia ≥ 100/μl. High sensitivity and specificity for the diagnosis of non-falciparum malaria are desirable; however there is no internationally agreed threshold.

### Criteria used to select the tests under evaluation in this study

The CareStart™ Malaria pLDH (Pan) test, a two-line test, shown to be reliable for the diagnosis of falciparum malaria was selected to validate its use in an area where non-falciparum infections were prevalent [13]. The WHO does not endorse particular tests but lists products available for procurement, which meet certain criteria e.g. manufactured to good manufacturing practice standards [14]. Since the time this study was performed, in order to be included in the procurement list, products must now also have been volunteered for the WHO product testing programme. The CareStart™ Malaria pLDH (Pf, Pan) three-line test was included in this study in the hope that the good performance of the two-line test might be reproduced with this test, and OptiMAL-IT^® ^was also included, since published results suggested it was one of the most sensitive RDTs for diagnosing non-falciparum species [15].

### Aims

The main aims of the study were to evaluate the sensitivity, specificity, positive and negative predictive values of the three RDTs for the detection of vivax and falciparum malaria compared to microscopy of Giemsa-stained blood slides, to study the time taken for positive tests to become negative, and to evaluate the inter-observer agreement, ease of use of the tests and heat stability.

Subsidiary aims included investigating differences in sensitivity of the tests in the under five year age group and the effect of parasitaemia or other covariates on sensitivity. In addition, sensitive PCR species detection was evaluated as an alternative reference standard in a subset of patients, which included all patients with positive slides, all patients with false positive RDT results compared to microscopy, and 20% of patients with negative slides selected at random.

### Study site and population

The study took place in a clinic where the non-governmental medical Organisation MSF-Switzerland works with the agreement of the Ministry of Health in Sonsinphya, Thayetchaung Township, Dawei in the south of Myanmar. The four Plasmodium species (falciparum, vivax, ovale and malariae), which commonly affect humans, are found here where malaria has a seasonal incidence, with a peak in the rainy season between June and August. The vast majority of cases (>80%) occur in patients over 5 years old.

### Ethical review

The study protocol was approved by the Comité de Protection des Personnes (CPP), Ile-de-France XI, St- Germain-en-Laye, France and local authorities in Myanmar gave their permission for the study to be implemented.

### Study design

A prospective, single-blind evaluation of three RDTs compared to slide microscopy.

### Sample size

Based on local 2006 data, when prevalences of Pf and Pv among fever cases were 30% and 10% respectively, it was estimated that 460 patients would be required to detect Pf with sensitivity and specificity of 90% (alpha error 0.05, precision 5%) and 960 patients would be required to detect Pv with the same sensitivity and specificity but a precision of 6% (N = 1383 with precision 5%).

The sample size was set at 1,000. The study was not powered to evaluate detection of mixed infections. A convenient sample size of 120 patients with a positive RDT result and positive malaria slide (60 Pf, 60 Pv) was chosen to describe the time taken for the RDTs to become negative.

### Informed consent

Patients were provided with an information sheet and the study was explained in their own language by the study personnel (Burmese or Karen). Written consent was obtained from participants or parent/guardian in the case of children.

### Inclusion and exclusion criteria

Main inclusion criteria were age> two years, with suspected malaria defined as fever (tympanic temperature>37.5°C), or a history of fever in the previous 48 hours, no signs of severity/danger signs. Exclusion criteria were pregnancy or having received a treatment course of antimalarials in the previous 4 weeks. To be eligible for inclusion into the follow-up study subjects needed to have a positive malaria slide for Pf or Pv mono-infection in conjunction with a positive RDT and to be able to attend follow-up until 28 days.

### Rapid diagnostic tests evaluated

CareStart™ Malaria pLDH (Pan) (AccessBio, New Jersey, US). A two-line test.

CareStart™ Malaria pLDH (Pan, Pf) (AccessBio, New Jersey, US). A three-line test.

OptiMAL-IT^® ^pLDH (Pan, Pf) (Diamed AG Switzerland). A three-line test.

The terms 'two-line' and 'three- line' are used to facilitate distinction between the two types of CareStart™ Malaria tests evaluated. The result of the CareStart™ Malaria two-line pLDH (Pan) was recorded as negative, positive or invalid. The CareStart™ Malaria three-line pLDH (Pan, Pf) and OptiMAL-IT^® ^test results were recorded as negative, Pf (+/- P.other), non-Pf or invalid. It should be noted that interpretation of the CareStart™ Malaria pLDH (Pan, Pf) test differs from OptiMAL-IT^® ^in that a test with a positive control line, positive Pf pLDH band and negative pLDH (Pan) should be interpreted as Pf. For OptiMAL-IT^® ^both Pf and pan pLDH bands must be positive to interpret the test as Pf.

### Blinding

Tests were labelled with the patient code on the underside. Two laboratory technicians performed the tests, took the capillary blood sample for haematocrit, the sample onto filter paper for PCR analysis and prepared the malaria blood slide. The slide was passed to another laboratory technician to stain and read, who was unaware of the RDT results. One test was handed to each of three readers for interpretation at the appropriate time. The tests were then handed on to three different readers, who were unaware of the first interpretation, to read and interpret the tests 10 minutes later. For the duration of the study each of the six readers read the same type of test and made the same reading each time (i.e. first or second).

### Visit schedule

All patients were seen on day 0 and the first 120 patients who agreed (60 with slide confirmed Pf mono-infection, 60 with Pv) were asked to return on days 2, 7, 14, 21 and 28 to document when the tests became negative. A malaria slide was performed simultaneously. Once the test was negative on any follow-up day they did not need to return for subsequent visits.

### Laboratory procedures

RDTs were performed according to the manufacturers' instructions. For all tests, a test without a control line was considered invalid and repeated. The number of invalid tests was recorded. Blood films were stained with 10% Giemsa for 20 minutes and read by experienced technicians. Parasite stages were counted separately by species. Trophozoites were counted on the thick (if count <500/500 WBC) or on the thin smear. At least 200 high power fields were examined before a slide was declared negative.

### PCR species sensitive detection

Sensitive species PCR genotyping on blood samples on filter paper was performed by the Shoklo Malaria Research Unit (SMRU) blind to the RDT and microscopy results. Parasite DNA was extracted from the bloodspot using the saponin lysis/chelex extraction method developed by Wooden and colleagues in 1993 [16]. DNA was stored at -20°C until processed. The targets of the *Plasmodium*-species PCR amplification are the genes coding for the small subunit ribosomal RNA (*ssRNA*). In the first amplification reaction (Nest 1), genus-specific primers were used to amplify a fragment of the *ssrRNA *genes of any *Plasmodium *parasite. The product of the first reaction was then used as the DNA template for a second amplification reaction (Nest 2). Species-specific primer pairs in the Nest 2 round amplified the specific sequence for *P. falciparum*, *P. vivax*, *P. malariae *or *P. ovale*. Primers and amplification conditions were those described by Snounou *et al *[17].

### Treatment

Patients with vivax malaria were treated with chloroquine 25 mg/kg divided over three days (10+10+5). Patients with falciparum malaria or mixed falciparum/vivax infections with a falciparum parasitaemia of < 4% infected red blood cells (irbc) were treated with mefloquine (25 mg/kg) + artesunate (4 mg/kg/day for three days)(MAS3). If the falciparum parasitaemia was ≥ 4% irbc they were treated with a seven-day regimen of artesunate (4 mg/kg for one day followed by 2 mg/kg/day for six days) and mefloquine (25 mg/kg) (MAS7). Those patients asked to come back for repeat testing to evaluate when the tests became negative had all doses of treatment supervised.

### Assessment of ease of use of tests

Ease of use of the tests was assessed using qualitative and quantitative criteria-*Qualitative*- laboratory technicians performing the tests were asked to rank the tests in order of preference where 1 corresponded to their most preferred test and 3 to their least preferred in each of the following categories - ease of taking blood, ease of adding reagents, ease of interpretation and overall performance.

*Quantitative *- number of steps in the procedure, time to wait before reading test.

### Quality assurance and quality control

The laboratory was already implementing internal quality control (QC) checks monthly and slides were sent periodically to an external laboratory (SMRU). In this study all slides were double-read, blind, by experienced technicians, with approximately seven days between the first and second readings. Slides with discordant results between two microscopists, defined as positive/negative discordance for asexual stages; species discordance for asexual stages; asexual parasite density discordance (difference in parasitaemia ≥ 50%) and gametocyte or malaria pigment reporting discordance were sent to SMRU for a third blind reading. The third reading was taken as the definitive result.

A sample of 120 of each of the CareStart Malaria™ tests (one box contains 60 tests) and 96 OptiMAL-IT^® ^tests (one box contains 24 tests) were sent to the Malaria RDT Quality Assurance Laboratory, Research Institute for Tropical Medicine, Muntinlupa City, Manila, Philippines for heat stability testing and quality control of performance according to Standard Operating Procedures (SOPs) under development by the WHO, Research Institute for Tropical Medicine, and others as part of a joint initiative to develop laboratory methods for malaria RDT assessment. Stability of the test result at one week in the field was also evaluated with a third blind reading made one week after the first to see whether retrospective checking of RDTs could be introduced as part of a regular QC procedure.

### Data management and analysis

Data from the case report forms was checked and entered on site in Microsoft^® ^Access. The source data forms with the RDT results were entered separately into Microsoft^® ^Excel. The two databases of RDT results were cross-checked. All discrepancies were corrected by returning to the source documents. Statistical analyses were performed using SPSS^® ^version 14.0 (Chicago, USA). Sensitivity, specificity, positive and negative predictive values of each test were calculated using microscopy as the reference standard. Sensitivity of the RDTs for detection of Pf and Pv mono-infections were then calculated. The first reading made of the RDT was used for these calculations. Sub-group analyses were performed to calculate the sensitivity and specificity of the tests at a parasitaemia below 100/μL and in the under five years age group. Multivariate analysis (logistic regression) was used to explore the relationship of selected covariates on test sensitivity and specificity (age, haematocrit, parasitaemia). Sensitivity and specificity of the tests and of slide microscopy compared to species PCR genotyping were also evaluated in a subset of patients. The confidence intervals (Wilson method) were calculated using Confidence Interval Analysis (CIA) software, version 21.2 (^©^Trevor Bryant 2002-2004, University of Southampton, UK). Agreement between the first and second and first and third readings of each RDT was assessed using the kappa coefficient.

### Definitions

Slides for which only gametocytes were detected were considered in two different ways, firstly as positive for that species, since gametocytes are known to produce pLDH, and secondly as negative since missing a solitary gametocytaemia is not usually an indication for treatment. Slides reported as showing malaria pigment only by microscopy were considered as negative.

## Results

**Figure 1:**
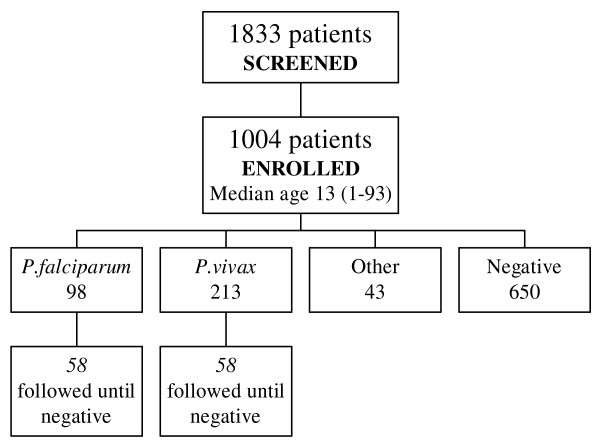
**Patient flow diagram**. Note: 'Other 'includes mixed infections and slides with either malaria pigment or gametocytes only. One subject <2 years enrolled (protocol violation).

Between 30^th ^August and 9^th ^November 2007, 1,004 patients were enrolled in the study out of a total of 1,833 patients attending the clinic with fever (Figure 1). The target sample size was exceeded in order to include 100 patients with falciparum malaria into the study. Reasons for non-inclusion of screened patients were not documented. Baseline characteristics of the included population are shown in Table 1. No serious adverse events were detected during the study. There were 13 protocol violations. One subject was less than two years of age, the lower limit for inclusion into the study. Eight patients with a parasitaemia ≥ PFT 40/1,000 irbc were treated with MAS3 instead of MAS7. Four patients were recruited into the follow-up phase of the study on the basis of a positive malaria smear result when their RDT result was negative and had to be excluded from the analysis.

**Table 1:** Baseline characteristics of included population

Male N (%)	517 (51.5)
Age in years	13 [1-93]

Weight in kg	30 [7-82]

Onset of symptoms in days	3 [1-20]

History of fever N (%)	1003 (99.9)

Temperature°C	37.2 [34.6-41.0]

Temp ≥ 37.5°C N (%)	420 (41.8)

Previous antimalarials N (%)	3 (0.3)

Haematocrit %	38(11-57)

Results expressed as median [range], unless otherwise stated

### Malaria slide results

The results of the slide microscopy on the day of enrolment (adjusted after quality control) are shown in Table 2. The sensitivity, specificity, positive and negative predictive values of the RDTs compared to slide microscopy are shown in Tables 3 and 4.

**Table 2:** Result of malaria slide microscopy for admission slides and all slides

**Final microscopy result**	**DAY 0** **N (%)**	**ALL SLIDES** **N (%)**
Negative	650 (64.7)	906(68.7)

PF trophozoites	98 (9.8)	113(8.6)
Parasitaemia/μL, geometric mean [range]	9618 [16-346737]	4688 [16-346737]

PV trophozoites	213 (21.2)	221(16.8)
Parasitaemia/μL, geometric mean [range]	2018 [16-107152]	1733 [16-107152]

PFT+PVT	6 (0.6)	7(0.5)

PMT	21(2.1)	21(1.6)

POT	7(0.7)	7(0.5)

Malaria Pigment	1(0.1)	17(1.3)

PF Gametocytes	2(0.2)	13(1.0)

PV Gametocytes	0	7(0.5)

Other^1^	6(0.6)	6(0.5)

^1 ^Other:

PVT, PVG, PFG 1/500 WBC

PFT 45/1000 RBC; PVG 2/500; MP

PFT 294, PFS 3/500 WBC, PMT 5, PMS 5, PMG 2/500 WBC, PVT 5, PVG 1/500 WBC

PVT 1, PVG 12, PVS 1/500 WBC, PMT 1, PMG 4/500 WBC

PFT 11, PMT 3, PMG 1, PVT 33, PVG 2/500 WBC, Very Rare MP

PFT 27/1000 RBC, PMT 50/500, PMG 70/500, PMS 8/500, MP

PF *P.falciparum*; PV *P.vivax*; PM *P malariae*; PO *P.ovale*; T = trophozoites

**Table 3:** Sensitivity, specificity, positive and negative predictive values of CareStart™ Malaria 2 line pLDH (Pan) results compared to microscopy results

	**Sensitivity**	**Specificity**	**Positive Predictive Value**	**Negative Predictive Value**
**PF/other^1^** (PF gametocytaemia considered negative for PF)	89.1 [85.5-91.8]	97.6 [96.5-98.4]	93.8 [90.8-95.9]	95.2 [94.2-96.8]

**PF/other^2^** (PF gametocytaemia considered positive for PF)	85.1 [81.3-88.2]	96.1 [94.7-97.2]	90.6 [87.3-93.2]	93.6 [91.9-95.0]

**PF mono-infection^1^** (PF gametocytaemia considered negative for PF)	95.6 [87.7-98.5]	-	-	-

**PF mono-infection^2^** (PF gametocytaemia considered positive for PF)	86.7 [79.9-91.4]	-	-	-

**PV mono-infection^1^** (PV gametocytaemia considered negative for PV)	91.0 [86.5-94.1]	-	-	-

**PV mono-infection^2^** (PV gametocytaemia considered positive for PV)	88.4 [83.7-91.9]	-	-	-

Note

1329 CareStart™ Malaria 2 line pLDH (Pan) tests performed; 13 (1%) were invalid, results were missing for 1 (0.1%) test., thus results of 1315 tests were evaluated.

Results expressed as % with 95% confidence intervals in brackets

Specificity not calculated for monoinfections since false positives for species individually cannot be estimated.

^1 ^Solitary gametocytaemia considered as a negative result for that species

^2^Solitary gametocytaemia considered as a positive result for that species

**Table 4:** Sensitivity, specificity, positive and negative predictive values of CareStart™ Malaria 3 line pLDH (Pan, Pf) and OptiMAL-IT^® ^results compared to microscopy

	**CareStart™ Malaria pLDH (Pan, PF)**				**OptiMAL-IT^®^**			
	**Sensitivity**	**Specificity**	**Positive Predictive Value**	**Negative Predictive Value**	**Sensitivity**	**Specificity**	**Positive Predictive Value**	**Negative Predictive Value**

**Pf +/- other^1^**	93.5 [87.8-96.7]	97.4 [96.3-98.2]	78.9 [71.6-84.7]	99.3 [98.7-99.7]	95.2 [89.8-97.9]	94.7 [93.3-95.8]	65.2 [58.0-71.8]	99.5 [98.9-99.8]

**Pf +/- other^2^**	89.1 [82.8-93.3]	98 [97-98.6]	83.7 [76.9-88.8]	98.7 [97.9-99.2]	91.8 [85.9-95.4]	95.4 [94.1-96.5]	69.5 [62.4-75.8]	99 [98.3-99.5]

**Non-Pf^1^**	78.5 [73-83.1]	97.8 [96.8-98.6]	89.5 [84.8-92.9]	95.1 [93.6-96.2]	90 [85.7-93.1]	96.5 [95.3-97.5]	85.9 [81.1-89.6]	97.6 [96.5-98.4]

**Non-Pf^2^**	77 [71.5-81.8]	97.9 [96.9-98.6]	90 [85.3-95.3]	94.6 [93.1-95.8]	90.2 [86-93.3]	97.1 [95.9-97.9]	88.2 [83.7-91.5]	97.6 [96.5-98.4]

**Pf mono-infection^1^**	94.7 [88.9-97.5]	-	-	-	94.7 [88.9-97.5]	-	-	-

**Pf mono-infection^2^**	90.5 [84.1-94.5]	-	-	-	92.1 [86-95.6]	-	-	-

**Pv mono-infection^1^**	80.6 [74.9-85.3]	-	-	-	91.8 [87.4-94.8]	-	-	-

**Pv mono-infection^2^**	78.9 [73.2-83.7]	-	-	-	92.1 [87.8-94.9]	-	-	-

Note

1337 CareStart™ Malaria 3 line pLDH (Pan, PF) tests performed; 21 (1.6%) were invalid, results were missing for 4 (0.3%) tests, thus results of 1312 tests were evaluated.

1328 OptiMAL-IT^® ^tests performed; 10 (0.8%) were invalid, results were missing for 1 (0.1%) test, thus results of 1317 tests were evaluated.

Results expressed as % with 95% confidence intervals in brackets

Specificity not calculated since false positives for species individually cannot be estimated.

^1 ^Solitary gametocytaemia considered as a negative result for that species

^2^Solitary gametocytaemia considered as a positive result for that species

### Effect of parasitaemia and age on sensitivity and specificity of RDTs

Sensitivities of the RDTs for detection of malaria at higher and lower parasitaemia were compared using 100 parasites/μL as the cut-off (Table 5). There were 113 *P. falciparum *and 218 *P. vivax *monoinfections recorded over the period of follow up with a geometric mean [range] parasitaemia of 4,688 [16-346,737]/μL and 1,733 [16-107,152]/μL respectively.

**Table 5:** Sensitivity of RDTs at a parasitaemia above and below 100/μL

	**Microscopy result**			
N	**PF <100/μL** 21	**PF ≥ 100/μL** 92	**PV <100/μL** 35	**PV≥ 100/μL** 183

**CareStart™ Malaria pLDH (Pan)**	76.2 [54.9-89.4]	100 [96-100]	45.7 [30.5-61.8]	99.5 [97.0-99.9]

**CareStart™ Malaria pLDH (Pan, PF)**	76.2 [54.9-89.4]	98.9 [94.1-99.8]	25.7 [14.2-42.1]	90.7 [85.6-94.1]

**OptiMAL-IT^®^**	81 [60-92.3]	97.8 [92.4-99.4]	62.9 [46.3-76.8]	97.3 [93.7-98.8]

95%CIs for the difference

C2 PF: 0.1-0.45; PV:0.375-0.690

C3 PF: 0.087-0.440; PV:0.479-0.770

OTM: PF: 0.043-0.379; PV: 0.20-0.51

The median [range] parasitaemias associated with a false negative RDT result were Pf 16/μL [16,16] (n = 5), Pv 16/μL [16-128] (n = 20) for CareStart™ Malaria two-line pLDH (Pan); Pf 16/μL [16,16] (n = 5), Pv 24/μL [16-288] (n = 30) for CareStart™ Malaria three-line pLDH (Pan, Pf) and Pf 16/μL [16-576] (n = 5), Pv 24/μL [16-48 230] (n = 14) for OptiMAL-IT^®^.

None of the patients with a presenting parasitaemia meeting the local definition of hyperparasitaemia (PFT ≥ 4% irbc) had a false negative RDT result. The number of patients under five years of age with positive slide results were small. Test sensitivities are shown in Table 6. In a multivariate analysis only the association between parasitaemia and RDT result was significant.

**Table 6:** Sensitivity, % [CI^95^] of the RDTs in the above and below 5 year age groups for the diagnosis of single-species infections compared to microscopy

**N**	**PF** **Age < 5 y** **12**	**PF** **Age ≥ 5 y** **101**	**PV** **Age < 5 y** **51**	**PV** **Age ≥ 5 y** **170**
CareStart™ Malaria 2 line pLDH (Pan)	100 [75.7-100]	95 [88.9-97.9]	88.2 [76.6-94.5]	91.8 [86.7-95.0]

CareStart™ Malaria 3 line pLDH (Pan, PF)	91.7 [64.6-98.5]	95.0 [88.9-97.9]	80.4 [67.5-89.0]	80.6 [74.0-85.8]

OptiMAL-IT^®^	91.7 [64.6-98.5]	95.0 [88.9-97.9]	92.2 [81.5-96.9]	91.7 [86.6-95.0]

95% CIs for the difference:

C2 PF: -0.112 to 0.194; PV: -0.046 to 0.156

C3: PF: -0.306 to 0.058; PV: -0.141 to 0.106

OTM: PF: -0.306 to 0.058; PV: -0.107 to 0.074

### Inter-observer agreement

Results for inter-observer agreement between the first and second readings of the tests performed 10 minutes apart and the first and third readings of the tests, performed one week apart are shown in Table 7.

**Table 7:** Inter-observer agreement for the 3 rapid diagnostic tests

	**1^st ^and 2^nd ^reading**		**1^st ^and 3^rd ^reading**	
	
	***N***	***kappa***	***N***	***kappa***
**CareStart™ Malaria 2 line pLDH (Pan)**	1315	0.967	1295	0.341

**CareStart™ Malaria 3 line pLDH (Pan, PF)**	1312	0.941	1258	0.280

**OptiMAL-IT^®^**	1316	0.923	1296	0.341

A kappa value of > 0.8 signifies very good agreement

2nd reading performed 10 minutes after the 1^st^; 3^rd ^reading performed 1 week after the 1^st^.

### Time for tests to become negative

Of 116 patients evaluated the median [range] time in days for the tests to become negative was 2 [2-14] for Pf monoinfections using both CareStart™ Malaria tests and 7 [0-21] for OptiMAL-IT^® ^and for Pv monoinfections was 2 [2-7] for CareStart™ Malaria 2 line pLDH (Pan), 2 [2-14] for CareStart™ Malaria three line pLDH (Pan, Pf) and 7 [0-14] for OptiMAL-IT^®^.

### Results of ease of use evaluation

General remarks made were that the Pf band of the CareStart™ Malaria three-line pLDH (Pan, Pf) test could be very faint making reading more difficult. For some tests the background was completely red, invalidating the test. It was important not to delay before adding the buffer since the tests could dry out quickly and give an invalid result. The information provided with the CareStart™ Malaria tests was unclear, the title of the packet inserts for both tests was the same and the two tests were in identical packaging, causing confusion. The pipettes for the CareStart™ Malaria tests were provided separately, unlike OptiMAL-IT^® ^where each test is pre-packed with the lancet, pipettes, alcohol swab and instructions. The buffers for the two tests were unlabelled. These points gave rise to a perception that the tests were of inferior quality to the OptiMAL-IT^®^. When it was very windy there were more invalid OptiMAL-IT^® ^tests because they tended to dry out between steps. The OptiMAL-IT^® ^pipette was found to be more difficult to use requiring twice as much blood as the CareStart™ Malaria tests. It was thought to be the most complicated test to perform, with 10 steps compared to only four for the CareStart™ Malaria tests. The CareStart™ tests were ranked joint first as the easiest to use.

### Quality assurance/Quality control results

#### Slide microscopy

From a total of 1,318 slides read, 228 (17%) were sent for a third blind reading. Of these 76 (5.7%) were sent because of differences in species between the first and second readers, 118 (9%) because of >50% variation in parasitaemia between first and second readings and the remainder because of discrepancies in recording of gametocytes or malaria pigment.

#### Rapid diagnostic test handling and quality control results

In July 2007, tests were sent from the manufacturers to the MSF logistic department in France by air and then onto Yangon. After one week in customs they were stored in a container at 20°C for two weeks. They were then transported to the central laboratory in Dawei, where the temperature varied between 12 and 24°C, and humidity from 62-81% (based on twice daily recordings). Tests were sent out to the field site in batches where the temperature varied between 23 and 31°C, humidity 50-90%.

#### Results of rapid diagnostic test temperature stability and in vitro QC testing

The CareStart™ Malaria two-line pan pLDH and CareStart™ Malaria three-line pan pLDH+Pf-specific pLDH were stable to 90 days at 35°C. For the CareStart™ Malaria three-line test, faint bands were observed on Pf (200 parasite/μl) samples on days 30, 60, and 90. The OptiMAL-IT^® ^Pf specific pLDH was stable up to day 20 at 35°C but failed stability testing beyond this point; the pan pLDH was stable up to day 90. All batches passed *in vitro *QC testing.

### Sensitive species PCR genotyping results

Sensitive species PCR genotyping was run on 662 enrolment and follow-up specimens. These included all slide or RDT positive samples and 20% of the negative samples selected at random. The results for the sensitivity and specificity of microscopy and the RDTs compared to PCR are shown in Tables 8 and 9. Table 10 provides an overall summary of the different attributes of the tests.

**Table 8:** Sensitivity and specificity of microscopy and CareStart™ Malaria 2 line pLDH (Pan) compared to sensitive species PCR genotyping

	**Microscopy**		**CareStart™ Malaria 2 line pLDH (Pan)**	
	**Sensitivity**	**Specificity**		

**Overall**	91.6 [88.3-94.0]	90.0 [85.7-93.1]	87.7 [84.1-90.6]	87.0 [82.4-90.6]

**PF monoinfection**	91.1 [84.8-95.0]	97.9 [96.2-98.8]	90.8 [84.6-94.6]	-

**PV monoinfection**	90.8 [86.3-94.0]	93.9 [91.2-95.8]	90.4 [85.7-93.6]	-

Note

PFG/PVG counted as a positive microscopy result for the purposes of this analysis

MP counted as negative

**Table 9:** Sensitivity and specificity of CareStart™ Malaria 3 line pLDH (Pan, PF) and OptiMAL-IT^® ^compared to sensitive species PCR genotyping

	**CareStart™ Malaria three line pLDH (Pan, PF)**		**OptiMAL-IT^® ^pLDH (Pan, PF)**	
	**Sensitivity**	**Specificity**	**Sensitivity**	**Specificity**

**PF +/- other**	86.0 [79.5-90.7]	95.9 [93.8-97.3]	92.6 [87.2-95.8]	91.4 [88.7-93.5]

**Non-PF**	77.3 [71.7-82.0]	93.6 [90.8-95.6]	89.2 [84.7-92.5]	90.2 [86.9-92.8]

**PF monoinfection**	78.6 [71.1-84.6]	-	91.5 [85.5-95.2]	-

**PV monoinfection**	78.4 [72.5-83.4]	-	90.3 [85.7-93.6]	-

**Table 10:** Summary table of test performance

	**CareStart™ Malaria 2 line pLDH (Pan)**	**CareStart™ Malaria three line pLDH (Pan, PF)**	**OptiMAL-IT^®^**
**PF detection^1 ^sensitivity % [CI^95^]**	95.6 [87.7-98.5]	94.7 [88.9-97.5]	94.7 [88.9697.5]

**PV detection^1 ^sensitivity % [CI^95^]**	91 [86.5-94.1]	80.6 [74.9-85.3]	91.8 [87.4-94.8]

**Heat stable at 90 days at 35°C**	Yes	Yes	No

**Ease of Use Ranking**	1	1	3

**Time to negative**, days	2	2	7

**Inter-reader agreement**	Excellent	Excellent	Excellent

**Cost (USD)**	0.6	1	1.5

**Invalid tests %**	1	1.6	0.8

^**1**^Compared to microscopy with presence of gametocytes regarded as a NEGATIVE result for that species

## Discussion

In this evaluation the OptiMAL-IT^® ^test was the most sensitive test for detection of malaria; however, this was at the expense of a slightly lower specificity than the CareStart™ Malaria tests, and hence, lower positive predictive value in this area, particularly for the detection of falciparum malaria or mixed falciparum/other infections.

The sensitivity of the CareStart™ Malaria pan pLDH test for the detection of all species combined was only 85.1% [CI^95 ^81.3-88.2]. However breaking this down to look at the detection of falciparum or vivax mono-infections only, sensitivity was considerably higher at 95.6 and 91.0% respectively. This is probably explained by the lower parasitaemia of the other infections. The CareStart™ Malaria pLDH (Pan, Pf) test was 93.5% sensitive for detection of falciparum-containing infections but performed poorly for the detection of non-falciparum infections with a sensitivity of only 78.5% [CI^95 ^73-83.1] which would limit its utility in areas with a high prevalence of non-falciparum infections.

How solitary gametocytemia was defined had a big impact on all the results, with an important decrease in sensitivity if a slide with Pf or Pv gametocytes, but no trophozoites was counted as positive for Pf or Pv rather than negative.

All three tests were highly sensitive for the detection of falciparum malaria monoinfections. For a parasitaemia > 100/μL all tests were >95% sensitive for the detection of Pf meeting the criteria set out by the WHO. This is extremely important from the point of view of patient safety. There were five missed falciparum infections, but these were all low parasitaemia infections, the highest being 36 Pf trophozoites/500 white blood cells on the thick smear.

Roll Back Malaria has only recently abandoned its recommendation that children under five years of age with fever in high transmission areas should be treated for malaria rather than performing a diagnostic test. There was no indication that the tests performed differently in younger age groups; however numbers of young children included were small.

The CareStart™ Malaria tests became negative rapidly at a median time of two days, a big advantage if deployed for diagnosis in high transmission areas, where reinfection is common. The OptiMAL-IT^® ^test took a longer time to become negative (seven days) although this is still faster than HRP-2 based tests.

Stability testing of the CareStart™ Malaria tests was satisfactory, but the OptiMAL-IT^® ^batch used in the study failed to demonstrate stability of the Pf pLDH band beyond 20 days at 35°C, which is poor, especially considering that these tests are used in tropical countries where temperatures above 35°C are common. Inter-observer agreement between readers was high for all tests but the result was not stable after a week (Table 7), thus in practice tests cannot be stored to review the results at a later date. With training the tests were easy to use but the number of steps to perform the OptiMAL-IT^® ^test meant it was fairly labour-intensive and not necessarily an ideal test for a crowded waiting room in a health post. The instructions provided with the CareStart™ Malaria three line pLDH (pan, Pf) test were a little confusing. They state that a test with a positive control line, positive Pf pLDH band and negative pLDH (Pan) should be interpreted as Pf. For OptiMAL-IT^® ^both Pf and pan pLDH bands must be positive to interpret the test as Pf. On contacting the manufacturers they explained that this is because the pLDH is sometimes completely used up by the Pf pLDH band.

It is usually assumed that PCR is more sensitive and specific for diagnosis than microscopy. Expert microscopy vs PCR would be expected to have specificity close to 100% [17]. In this study, specificity for detection of Pv was lower than for Pf. Possible explanations are inaccurate microscopy (despite the QA/QC measures in place), sub-optimal sensitivity of the PCR method or probably a combination of the two. The advantage of using expert microscopy over PCR sensitive species genotyping is that it gives information about parasite stage and parasitaemia. PCR may be a good alternative in areas where expert microscopy cannot be put in place for a study or in travellers returning to non-endemic areas. In an endemic area PCR may detect an infection that has cleared below the limit if microscopic detection or has been treated. Surprisingly, only three subjects (0.3%) admitted to having taken antimalarials before presenting to the clinic.

A large number of patients presenting with fever or history of fever (64.7%) did not have malaria. This low prevalence was reflected in the relatively low PPV of the OptiMAL-IT^® ^test of 69.5% [95%CI 62.4-75.8] for falciparum-containing infections. Malaria is not the only disease for which improved diagnostics are needed at field level. Leptospirosis and rickettsial diseases, such as scrub and murine typhus, are common in SE Asia. Describing the epidemiology of fever in this population would lead to improved case management. Among those who did have malaria, the vivax prevalence was more than double that of falciparum, a reversal of the ratio observed in 2006 in the same area. The explanation for this is unclear since ACT has been available to treat falciparum malaria in this area for some years. However, access may have improved in adjacent areas as a large donation of artemether-lumefantrine was deployed in government clinics across Myanmar in 2007 with a consequent impact on transmission of falciparum malaria.

The OptiMAL-IT^® ^was highly sensitive for the detection of falciparum and vivax malaria and would perform best in an area of high malaria prevalence among screened fever cases. However heat stability was not acceptable and the number of steps to perform this test is a significant drawback in the field.

The CareStart™ Malaria pLDH (Pan) test may be a good alternative to Paracheck-Pf™ in areas where the predominant species is *P. falciparum*, particularly if transmission is high since it becomes negative rapidly.

Heat stability remains a major concern for the pLDH tests and stability testing at intervals as part of quality assurance and quality control of malaria diagnostic procedures is recommended in programmes using pLDH based RDTs. Unless manufacturers provide convincing data on heat stability from different test batches, a rapid diagnostic test should not be recommended.

Choosing a rapid diagnostic test to deploy in the field depends on numerous factors. High sensitivity and specificity for detection of disease are the most important features of a good test; however these become less relevant if the test is not heat-stable in field conditions, if the test is too complicated to perform or if inter-observer agreement is poor. Cost is also a factor, but costs may vary greatly depending on the quantity ordered and a recent increase in the number of tests on the market should lead to prices coming down.

The drawbacks of the HRP-2 tests have been described; however it could be argued that these are outweighed by their high sensitivity for detection of potentially life-threatening falciparum malaria and their superior heat stability.

The approach used here to select tests at random and evaluate them is expensive and labour intensive. The results of the first round of the WHO product testing have been published recently. In general, highest Pf detection rates were demonstrated by tests targeting HRP2. Batch-to-batch variation was also observed leading to the recommendation to test new lots post purchase and prior to use [18]. This initiative by the WHO to centralize RDT evaluations and perform *in vitro *QC and stability testing is welcomed as this should accelerate the process of getting much needed reliable rapid diagnostic tests in the field.

## Competing interests

The authors declare that they have no competing interests.

## Authors' contributions

EA, PJG, FN, SP, A-LP, RH, MT, MA and EC conceived of the study, participated in its design and coordination and helped to draft the manuscript. EA analysed the data and wrote the first draft of the manuscript. CD and JL performed the stability testing, analysed these data and wrote part of the manuscript. KH, MML, ML, PH and AK collected data and revised the manuscript. All authors read and approved the final manuscript.
